# Loss of Smek1 Induces Tauopathy and Triggers Neurodegeneration by Regulating Microtubule Stability

**DOI:** 10.1002/advs.202400584

**Published:** 2024-08-29

**Authors:** Ruo‐Nan Duan, Ai Liu, Yue‐Qing Sun, Yun‐Fang Xie, Shi‐Jun Wei, Shang Gao, Yi‐Ming Liu, Xi Li, Wen‐Jie Sun, Jiang‐Xia Li, Chuan‐Zhu Yan, Qi‐Ji Liu

**Affiliations:** ^1^ Department of Neurology, Research Institute of Neuromuscular and Neurodegenerative Disease, Qilu Hospital, Cheeloo College of Medicine Shandong University No. 107 West Wenhua Road Jinan Shandong 250012 China; ^2^ Key Laboratory for Experimental Teratology of the Ministry of Education and Department of Medical Genetics, School of Basic Medical Sciences Shandong University No. 44 West Wenhua Road Jinan Shandong 250012 China; ^3^ Mitochondrial Medicine Laboratory, Qilu Hospital (Qingdao), Cheeloo College of Medicine Shandong University Qingdao Shandong 266000 China; ^4^ Key Laboratory for Experimental Teratology of the Ministry of Education and Department of Medical Genetics, School of Basic Medical Sciences, Shandong University, School of Health and Life Sciences University of Health and Rehabilitation Sciences Qingdao 266071 China

**Keywords:** Kif2a, neurodegeneration, Smek1, tau phosphorylation, tauopathy

## Abstract

Suppressor of Mek1 (Smek1) is a regulatory subunit of protein phosphatase 4. Genome‐wide association studies have shown the protective effect of *SMEK1* in Alzheimer's disease (AD). However, the physiological and pathological roles of Smek1 in AD and other tauopathies are largely unclear. Here, the role of Smek1 in preventing neurodegeneration is investigated in tauopathy. Smek1 is downregulated in the aged human brain. Through single‐cell sequencing, a novel neuronal cluster is identified that possesses neurodegenerative characteristics in Smek1^−/−^ mice. Smek1 deficiency caused markedly more severe motor and cognitive impairments in mice, as well as neuronal loss, gliosis, and tau hyperphosphorylation at major glycogen synthase kinase 3β (Gsk3β) sites. Protein‐protein interaction analysis revealed that the Ran‐binding domain (RanBD) in the N‐terminus of Smek1 facilitated binding with kinesin family member 2A (Kif2a). Depletion of Smek1 resulted in cytoplasmic aggregation of Kif2a, axon outgrowth defects, and impaired mitochondrial axonal trafficking. Downregulation of Kif2a markedly attenuated tau hyperphosphorylation and axon outgrowth defects in shSmek1 cells. For the first time, this study demonstrates that Smek1 deficiency progressively induces neurodegeneration by exacerbating tau pathology and mitochondrial dysfunction in an age‐dependent manner.

## Graphical abstract

Impairment in microtubule stability and mitochondria transportation is a common pathway in neurodegeneration. The present study elucidates a mechanism by which Smek1 protects against tauopathy by binding to Kif2a in neurons and thereby regulates tau phosphorylation. Smek1 deficiency leads to declined motor and cognition function in mice. Together, they identify Smek1 as a novel therapeutic target in tauopathy.

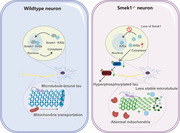

## 1 Introduction

Tauopathies are a group of neurodegenerative diseases characterized by hyperphosphorylation of the microtubule‐binding protein tau and typically affect microtubule stability and axoplasmic transport.^[^ ^1^ ^]^ Mutations in the Microtubule‐associated protein tau (*MAPT*) gene are causative of various tauopathies. Genome‐wide association studies (GWASs) have described several susceptibility genes for Alzheimer's disease (AD)^[^ ^2^ ^]^ and the shared risk variants in frontotemporal dementia (FTD),^[^ ^3^ ^]^ progressive supranuclear palsy (PSP) and corticobasal degeneration (CBD).^[^ ^4^ ^]^ However, the mechanisms by which the genetic factors derived from GWAS‐hit genes modulate disease risk remain largely unclear.

Suppressor of Mek1 (Smek1, also known as PPP4R3A), a regulatory subunit of serine/threonine‐protein phosphatase 4 (PP4), is a conserved protein that participates in various biological processes.^[^ ^5^ ^]^ A GWAS indicated that *PPP4R3A* rs2273647‐T is related to disease risk and progression in Alzheimer's disease.^[^ ^6^ ^]^ The authors determined that the minor allele (T) dosage was significantly associated with a reduced probability of developing mild cognitive impairment (MCI) or AD and modified the extent of cognitive decline over time. Smek1 is localized in the nucleus, perinucleus, and cytoskeleton.^[^ ^7^ ^]^ In fact, its subcellular localization depends on cell cycle phases. Smek1 is located in the nucleus during interphase and prophase and is transported to the cytoplasm and mitotic spindle from prometaphase to anaphase. The above dynamic distribution changes rely on the nuclear localization sequence (NLS) at the carboxyl terminus of the Smek1 protein.^[^ ^5d^ ^]^ A previous study revealed that Smek1 could determine the subcellular localization of protein phosphatase 4 catalytic subunit (PP4C).^[^ ^8^ ^]^ In the nervous system, Smek1 is required for neurogenesis by regulating neural stem/progenitor cell differentiation in the embryonic stage.^[^ ^5b,d,e^ ^]^ Studies have also demonstrated that Smek1 is involved in senility. Loss of Smek1 in *C. elegans* resulted in shortened life expectancy.^[^ ^9^ ^]^ Recent results indicated that Smek1 mediates stress resistance and longevity in *C. elegans* by regulating the transcriptional initiation of DAF‐16‐activated genes.^[^ ^10^ ^]^ In the current study, we address the role of Smek1 in triggering tauopathy and neurodegeneration.

## 2 Results

### 2.1 Transcriptional Level of Smek1 is Reduced in Aging Brains

**Figure 1:**
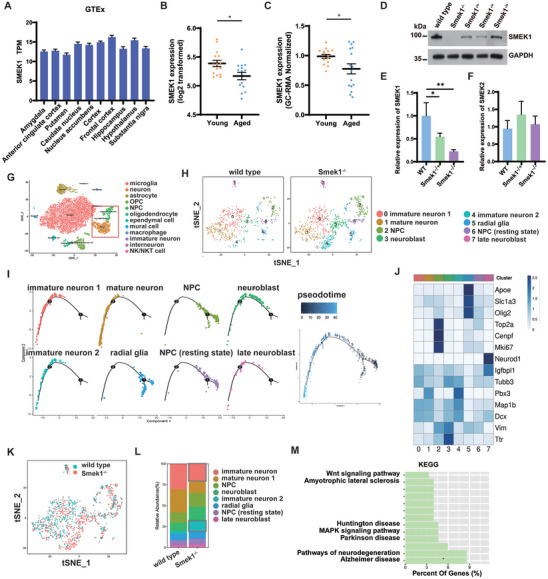
Suppressor of Mek1 (Smek1) deficiency is associated with neurodegeneration. A) GTEx database revealed wide expression of SMEK1 among different regions in human brain. Amygdala, n = 100; Anterior cingulate cortex, n = 121; putamen, n = 124; caudate nucleus, n = 160; nucleus accumbens, n = 147; cortex, n = 158; frontal cortex, n = 129; hippocampus, n = 123; hypothalamus, n = 121; substantia nigra, n = 88. B) Transcription level of SMEK1 in human prefrontal cortex (n = 15,14) between young (20‐44 yrs) and aged (86‐106 yrs) group. C) Transcription level of SMEK1 in human hippocampus (n = 15,16) in young (20‐45 yrs) and aged (80‐99 yrs) group. D) Western blot analysis of Smek1 knockout efficiency. E) QPCR analysis of Smek1 knockout efficiency. N = 4 per group. F) QPCR analysis of Smek2 expression level. N = 4 per group. G) TSNE visualization of individual cell clusters from the cortex and hippocampus of wild‐type (n = 2) and Smek1^−/−^ (n = 2) mice. H) TSNE map of neuron and stem cells in wild type and Smek1^−/−^. I) Pseodotime analysis of eight clusters revealed differentiated states of each cluster. J) Normalized mean expression of neural markers across all eight clusters (cl). Clusters have been coded by numbers (at the bottom) and by color (at the top). These correspond to the numbers and colors shown in (H). K) Contribution of wild‐type versus Smek1^−/−^ mouse samples to eight clusters. (L) The proportion of each cluster among wild type and Smek1^−/−^ mouse samples. M) Kyoto Encyclopedia of Genes and Genomes (KEGG) pathway analysis of marker genes in cluster 4. Data are mean ± SD and are analyzed by two‐sided unpaired t test; ^*^ *p* < 0.05; ^**^ *p* < 0.01.

To obtain an overall view of Smek1's spatial characteristics in different brain regions, open access data in GTEx were analyzed. The transcription profile of Smek1 showed a widely expressed pattern in different brain regions (**Figure** **1**A). To reveal the potential age‐related characteristics of Smek1 in the brain, we analyzed mRNA expression data from the prefrontal cortex (PFC) (GSE53890)^[^ ^11^ ^]^ and hippocampus (GSE11882).^[^ ^12^ ^]^ SMEK1 expression in the PFC (Figure 1B) and hippocampus (Figure 1C) was compared between young and aged cases. Smek1 was found to be downregulated in the PFC and hippocampus of aged cases.

To investigate the characteristics of Smek1 in neurodegeneration, we generated Smek1 constitutive knockout mice. In Smek1^−/−^ mouse brain tissue homogenate, Smek1 protein was barely detected by Western blotting in brain tissue (Figure 1D). The qPCR results showed that ≈50% and 20% of the Smek1 mRNA remained in heterozygous and homozygous mouse brain tissue, respectively (Figure 1E). Notably, suppressor of mek1 homolog 2 (Smek2) was retained (Figure 1F). Single‐cell RNA sequencing was carried out on 2‐month‐old mice as previously reported (Figure 1G).^[^ ^13^ ^]^ To define the neuron type, we performed subclustering analysis of neural progenitor cells, immature neurons, and neurons. The neuronal cells were classified into radial glia, neural progenitor cells, neuroblasts, late neuroblasts, immature neurons, and mature neuron in the wild‐type and Smek1^−/−^ mice (Figure 1H). Analysis of the median expression of known neuronal marker genes confirmed successful clustering of cells by subtype (Figure 1J). Among the above clusters, two immature neurons were defined. Pseudotime analysis predicted that both clusters were in similar cell fate (Figure 1I). However, immature neuron 2 mostly existed in Smek1^−/−^ mice (Figure 1K,L). Enrichment analysis of immature neuron 2 showed that immature neuron 2 is associated with several neurodegenerative diseases, i.e., amyotrophic lateral sclerosis, Huntington disease, Parkinson's disease, and Alzheimer's disease (Figure 1M). The existence of these unique neurons in Smek1^−/−^ mice suggested that Smek1 deficiency causes an early neurodegeneration. Thus far, the above evidence indicates that Smek1 is likely to play a pivotal role in maintaining brain functions and that loss of Smek1 may contribute to age‐related neurodegeneration.

### 2.2 Smek1^−/−^ C57BL/6 Mice are Substantially Embryonically Lethal

**Figure 2:**
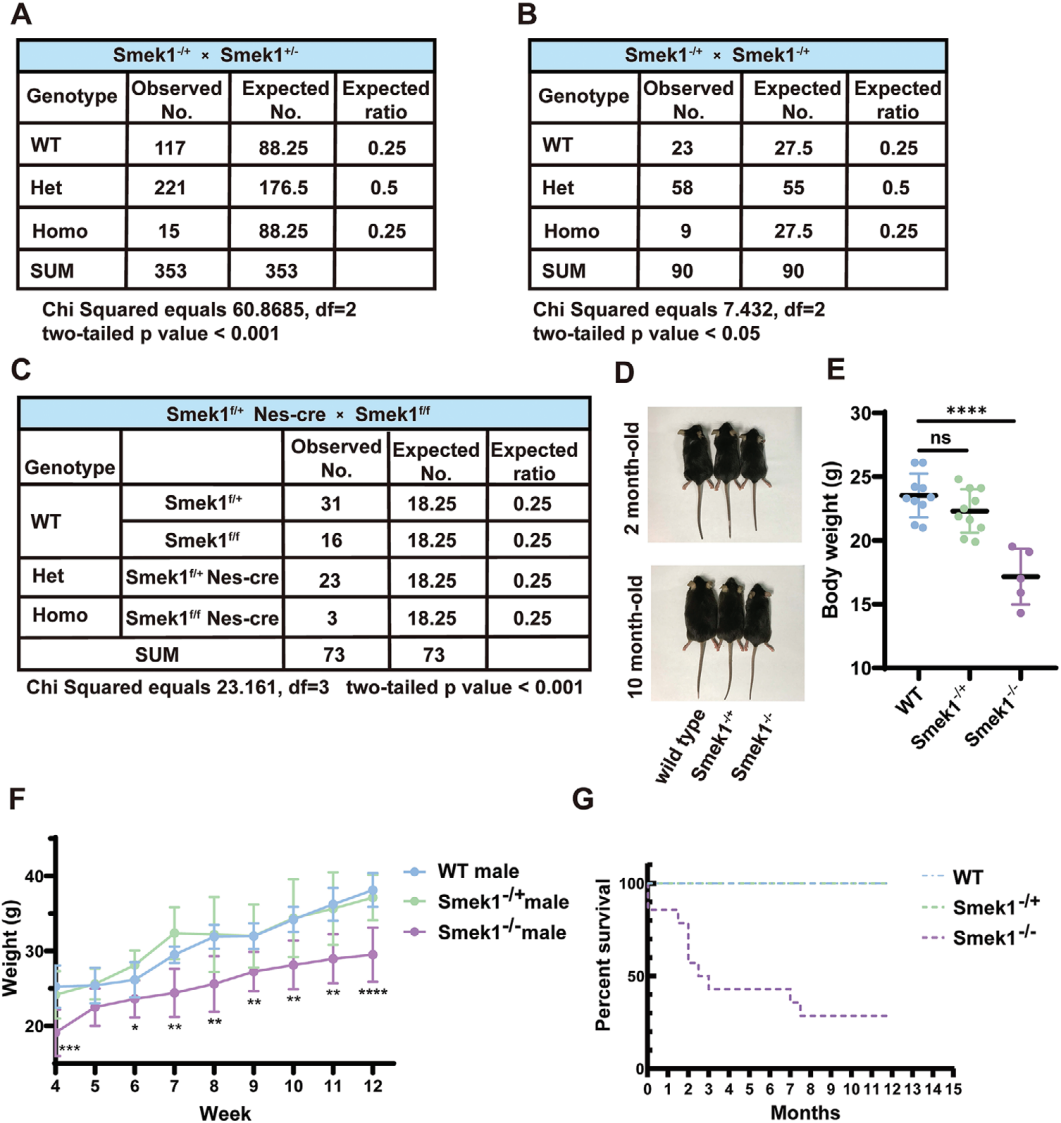
Smek1‐deficient mice survive but show several deficits. A) Summary of genotypes observed in P21 following C57BL/6 Smek1^−/+^ mice cross mating. B) Summary of genotypes observed in P21 following ICR‐C57BL/6 Smek1^−/+^ mice cross mating. C) Summary of genotypes observed in P21 following C57BL/6 Smek1^f/f^ mice cross mating Smek1^f/+^ Nes‐Cre. D) Appearance of 2‐month‐old male mice and 10‐month‐old male mice. E) Body weight of 2‐month‐old male mice (C57BL/6 strain) (N = 5‐10). F) Body weight recorded in 4‐week to 12‐week‐old male mice (ICR‐C57BL/6 strain) (N = 5‐12). G) Kaplan Meier survival curves of observed postnatal mice (C67BL/6 strain). Data are mean ± SD and are analyzed by two‐sided unpaired t test; ^*^ *p* < 0.05; ^**^ *p* < 0.01; ^***^ *p* < 0.001; ^****^ *p* < 0.0001.

Since the features of Smek1 knockout mice after birth have not been reported, we first focused on the details of the biological function of Smek1 at the individual level in mice. The genotype ratio between wild‐type, heterozygous, and homozygous mice deviated from the expected ratio, despite the repeated crossing of Smek1^−/+^ mice (**Figure** **2**A). Previous studies have shown that changing the genetic background by mating the inbred C57BL/6 strain with the outbred ICR strain could rescue prenatal/postnatal lethality in gene knockout mice.^[^ ^14^ ^]^ We generated mixed background mice by mating C57BL/6 mice with ICR mice (Figure S1A,B, Supporting Information). Compared to the C57BL/6 strain, the number of homozygotes with a mixed genetic background slightly increased but still deviated from Mendel's law (Figure 2B). Next, to elucidate whether Smek1 depletion in the central nervous system is a causative factor in prenatal death, we generated Smek1 conditional knockout C57BL/6 mice driven by Nes‐Cre (Figure S1C, Supporting Information). The Smek1^f/f^ Nes‐Cre number also deviated from the expected number (Figure 2C). Furthermore, C57BL/6 (Figure 2D,E), mixed background Smek1^−/−^ (Figure 2F), and Smek1^f/f^ Nes‐Cre (Figure S1D, Supporting Information) mice all exhibited a reduction in body weight. The body weight of homozygous mice exhibited slow growth. Strikingly, a majority of the observed C57BL/6 homozygous mice suffered from untimely death, and only a few mice could live up to the age of the wild‐type mice (Figure 2G). The subsequent experiments were carried out using constitutive knockout mice with a C57BL/6 background. Overall, these results suggested that substantial loss of Smek1 resulted in abnormal development in embryonic and premature stages, as well as shortened life span.

### 2.3 Smek1‐Deficient Mice Display Behavioral Phenotypes Associated with Tauopathy in an Age‐Dependent Manner

**Figure 3:**
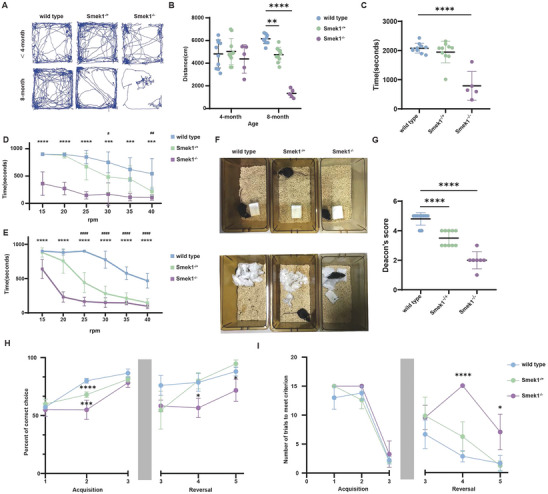
Smek1 knockout mice showed declination in locomotion and cognition ability. A) Mice movement tracks in open field test. N = 10 in wild‐type and Smek1^−/+^ and N = 5 in Smek1^−/−^. B) Locomotion distance in open field test. N = 10 in wild‐type and Smek1^−/+^ and N = 5 in Smek1^−/−^. C) Treadmill test of 6‐month‐old mice. N = 10 in wild‐type and Smek1^−/+^ and N = 5 in Smek1^−/−^. D) Rotarod test of 6‐month‐old mice. N = 10 in wild‐type and Smek1^−/+^ and N = 5 in Smek1^−/−^. ^*^, p value compared between wild type and Smek1^−/−^; # p value compared between wild type and Smek1^−/+^. E) Rotarod test of 10‐month‐old mice. N = 10 in wild‐type and Smek1^−/+^ and N = 5 in Smek1^−/−^. ^*^, p value compared between wild type and Smek1^−/−^; # p value compared between wild type and Smek1^−/+^. F,G) Nesting behavior and Deacon's score of 6‐month‐old mice. N = 10 in wild‐type and Smek1^−/+^ and N = 7 in Smek1^−/−^. H) Percent of correct choice in Water T‐maze of 6‐month‐old mice during acquisition learning phase (day 1–3) and reversal learning phase (day 3–5). N = 8‐10 per group. Data are mean ± SD and are analyzed by two‐sided unpaired t test; ^*^ *p* < 0.05; ^**^ *p* < 0.01; ^***^ *p* < 0.001; ^****^ *p* < 0.0001.

We performed a series of behavioral tests to assess the condition of knockout mice on a C57BL/6 background (Figure S1E, Supporting Information). We first performed the open field test to measure physical/motor activity. The results showed reduced total distance traveled in 8‐month‐old Smek1^−/−^ mice but not in mice before 4 months of age (**Figure** **3**A,B). The treadmill test of 6‐month‐old male mice revealed slower exercise endurance in homozygous mice (Figure 3C). Decreased duration in the rotarod test was observed in 6‐month‐old (Figure 3D) and 10‐month‐old (Figure 3E) homozygous mice. Notably, compared to wild‐type mice, exercise endurance declined more rapidly in heterozygous mice from 6 months old to 10 months old. Nesting behavior was assessed according to Deacon's score^[^ ^15^ ^]^ in 6‐month‐old mice. Wild‐type mice were capable of building nearly perfect nests, while pressed cotton in heterozygous and homozygous cages was partially torn (Figure 3F,G).

To evaluate cognitive ability, a T‐maze was performed on 6‐month‐old mice that showed cognitive deficiency in homozygous mice. During the acquisition learning phase, the Smek1^−/+^ and Smek1^−/−^ groups had lower percentages of correct choices on days 2 and 3. The Smek1^−/−^ group also had a lower percentage of correct choices in the reversal learning phase (Figure 3H). There was no significant difference among groups in the number of trials needed to reach the acquisition criterion. However, on days 4 and 5, the Smek1^−/−^ group had more attempted trials (Figure 3I). In brief, motor disability and cognition decline were observed with age in Smek1 KO mice, especially in homozygotes.

### 2.4 Neuronal Loss and Gliosis in Smek1‐Deficient Aged Mice

**Figure 4:**
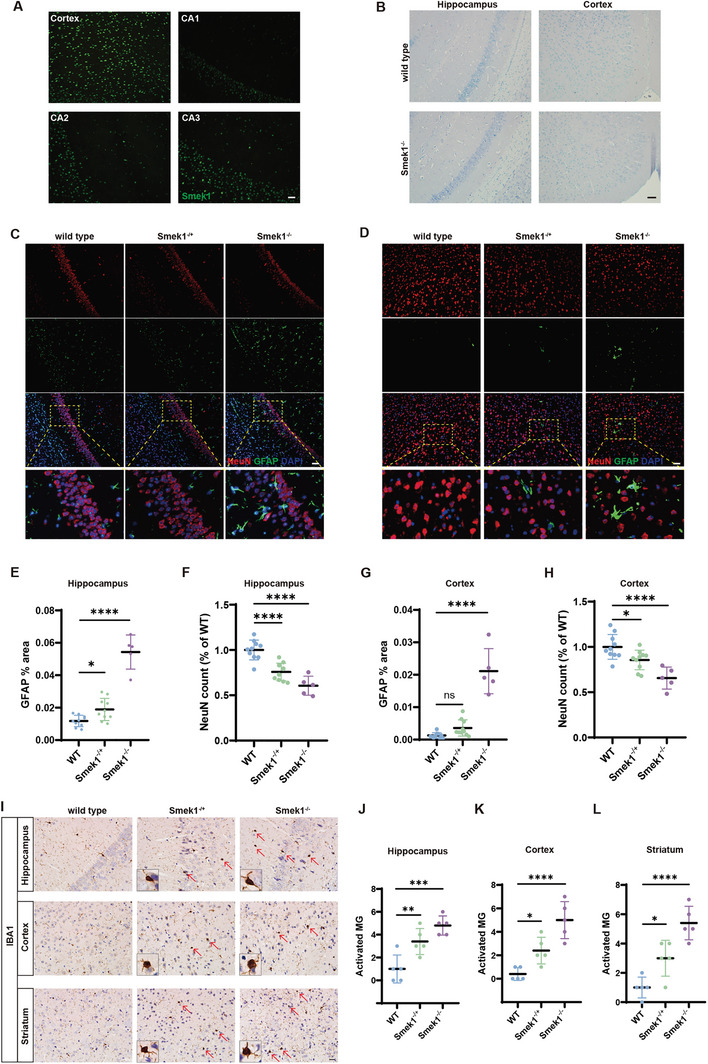
Smek1 deficiency causes neurodegeneration in mice. A) Smek1 staining in mice prefrontal cortex and different regions of hippocampus. Scale bar, 50 µm. B) Nissl staining of 2‐month‐old CA1 hippocampus and motor cortex. Scale bar, 50 µm. C) Co‐staining of NeuN and GFAP in 12‐month‐old hippocampus (CA1). Scale bar, 50 µm. D) Co‐staining of NeuN and GFAP in 12‐month‐old motor cortex. Scale bar, 50 µm. E–F) Intensity of fluorescent NeuN and GFAP staining in wild type, Smek1^−/+^ and Smek1^−/−^ mice. The average intensity was analyzed from images of immunostained cortex (N = 5–10). G–H) Intensity of fluorescent NeuN and GFAP staining in wild type, Smek1^−/+^ and Smek1^−/−^ mice. The average intensity was analyzed from images of immunostained hippocampus (N = 5‐10). (I) IBA‐1staining in 12‐month‐old hippocampus (CA1), motor cortex and striatum. Scale bar, 20 µm. J–L) Cell count of activated microglia (MG) in wild type, Smek1^−/+^ and Smek1^−/−^ mice. The average count was analyzed from images of immunostained hippocampus (J), cortex (K), and striatum (L). (N = 5). Data are mean ± SD and are analyzed by two‐sided unpaired t test; ^*^ *p* < 0.05; ^**^ *p* < 0.01; ^***^ *p* < 0.001; ^****^ *p* < 0.0001.

To verify our phenotypic findings, neurodegenerative‐related staining was carried out in aged mice (12 months old). We observed that Smek1 was expressed in both the cortex and hippocampus (**Figure** **4**A). At the age of 2 months, the number and arrangement of hippocampal neurons did not differ between the homozygous mice and wild‐type mice (Figure 4B). At the age of 12‐month, an increased number of astrocytes was observed in the hippocampus of the homozygous mice (Figure 4C,E). NeuN staining of the hippocampus revealed neuronal loss in the pyramidal cell layer of the homozygotes, accompanied by a loose arrangement of the remaining neuronal cells (Figure 4C,F). Loss of neurons and increased astrogliosis were also observed in the cortex (Figure 4D,G,H). IBA‐1 staining revealed increased numbers of activated ameboid microglia (Figure 4I) in hippocampus (Figure 4J), cortex (Figure 4K), and striatum (Figure 4L). Myelin basic protein (MBP) staining showed no apparent demyelination (Figure S2A, Supporting Information). Based on these findings, we suggest that Smek1 deficiency in mice leads to increased gliosis and loss of neurons in the central nervous system at a relatively young age.

### 2.5 Smek1 Deficiency Enhanced Tau Phosphorylation and Interfered with Microtubule Stability

**Figure 5:**
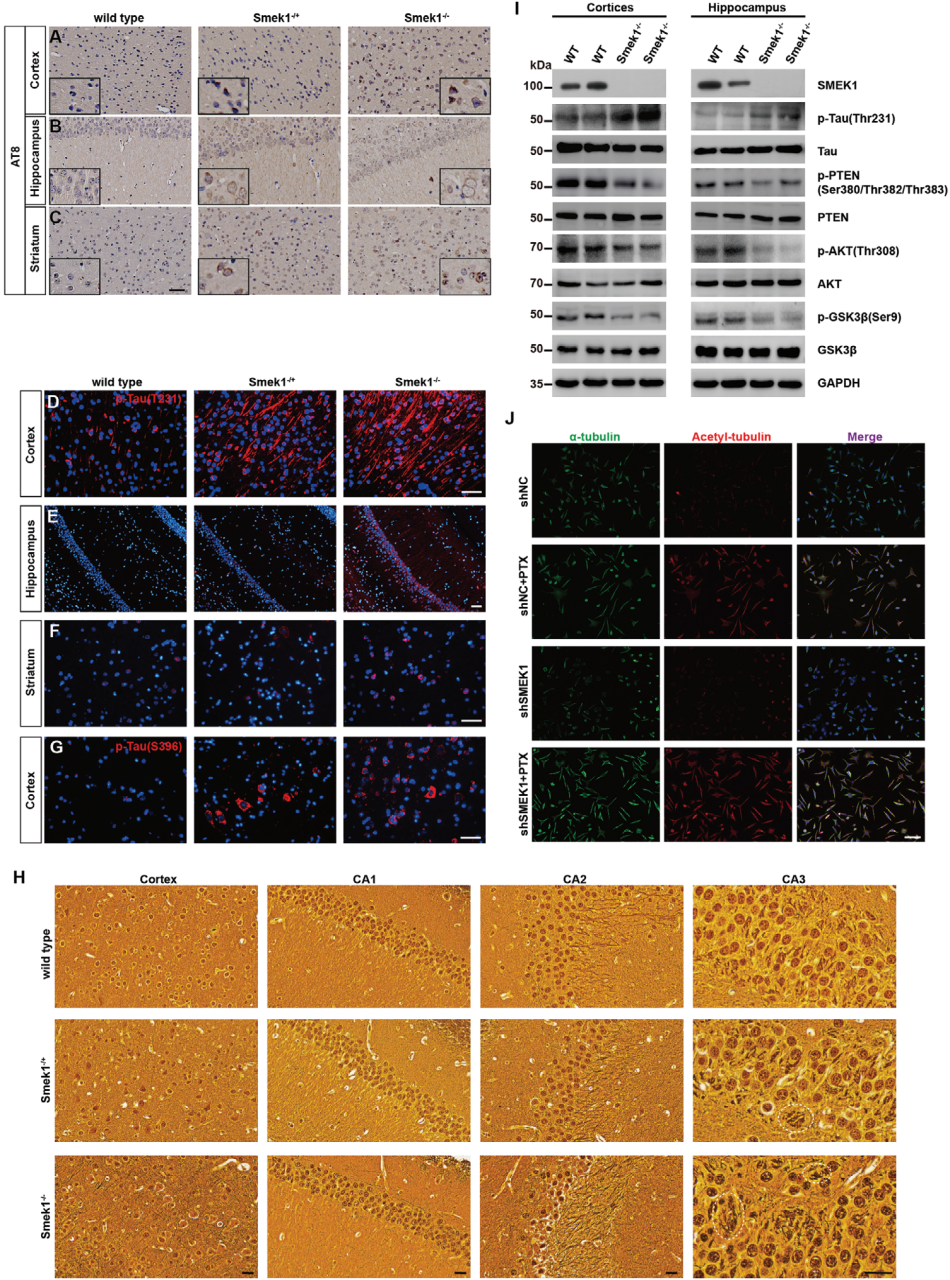
Smek1 deficiency enhances the development of tau pathology and interfere microtubule stability via PI3K/AKT pathway. AT8 labeling of the A) cortex, B) hippocampus, and C) striatum in 12‐month‐old brain. P‐Tau(T231) staining of D) hippocampus, E) cortex, and F) striatum in 12‐month‐old brain. Scale bar, 50 µm. P‐Tau(S396) staining of cortex G) in 12‐month‐old brain. Scale bar, 50 µm. H) Bielschowsky silver staining of hippocampus (CA1, CA2, and CA3) and motor cortex in 12‐month‐old brain. Dotted circle, NFTs. Scale bar, 25 µm. I) Western blot of brain tissue in 12‐month‐old wild type and Smek1^−/−^ mice. J) Co‐staining of α‐tubulin and acetyl‐tubulin of transfected SH‐SY5Y cells treated with or without Paclitaxel (PTX). Scale bar, 100 µm.

We next examined whether Smek1 deficiency could promote tau aggregate formation in the brain. Immunohistochemistry of AT8 indicated increased phosphotau‐positive neuron density in the cortex, hippocampus, and striatum (**Figure** **5**A–C). Moreover, p‐Tau(T231) (Figure 5D–F) deposition was increased in the cortex, hippocampus, and striatum of both Smek1^−/+^ and Smek1^−/−^ mice. We also observed an increased in the intensity of p‐Tau(S396) (Figure 5G) in the cortex of Smek1^−/+^ and Smek1^−/−^ mice. Neurofibrillary tangles (NFTs) were observed in the CA3 regions of Smek1^−/−^ mice and axonal degeneration was observed in hippocampus and cortex of Smek1^−/+^ and Smek1^−/−^ mice (Figure 5H). To confirm the presence of tau hyperphosphorylation and its upstream pathways, we carried out Western blotting analysis of the cortex and hippocampus. The results showed p‐PTEN, p‐AKT, and p‐GSK3β were downregulated, while p‐Tau (T231) was hyperphosphorylated in the Smek1^−/−^ group (Figure 5I).

Since tau hyperphosphorylation may cause impairment in microtubules, we examined microtubule morphologic changes in Smek1‐deficient nerves. Bielschowsky staining of the corpus callosum showed a loss of fibers in heterozygous and homozygous mice (Figure S2C, Supporting Information). In addition, Smek1 was detected in the sciatic nerve and colocalized with α‐tubulin (Figure S2B, Supporting Information). Electron microscopy of aged murine optic nerves (Figure S2D, Supporting Information) and sciatic nerves (Figure S2E,F, Supporting Information) revealed sparse microtubule distribution. In SH‐SY5Y cells, silencing Smek1 resulted in shorter neurites while overexpressing Smek1 had the opposite effect (Figure S2G, Supporting Information). Immunostaining of α‐tubulin and acetyl‐α tubulin showed less stabilized microtubules in shSmek1 cells, while microtubules extended after treatment with Taxol (Figure 5J). These results indicated that loss of Smek1 may increase p‐Tau levels via the AKT‐GSK3β pathway and cause loss of axons due to microtubule destabilization.

### 2.6 Smek1 Interacts with and Determines Kif2a Subcellular Location

**Figure 6:**
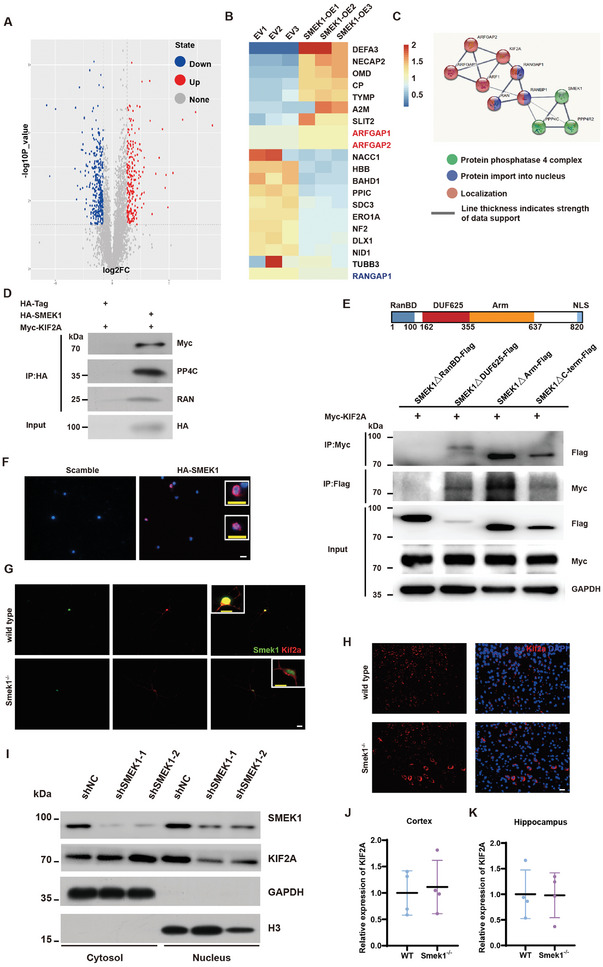
Smek1 interacts with kinesin family member 2A (Kif2a), while Smek1 deficiency caused cytoplasmic Kif2a aggregation. A) Volcano map of differentially expressed genes (DEGs) in proteomics analysis of SMEK1‐overxpressing and control SH‐SY5Y cells. B) Proteomics heatmap of SMEK1overxpressed SH‐SY5Y cells. C) Predicted interaction network between Kif2a and Smek1 in STRING database. D) Co‐immunoprecipitation (CoIP) analysis of HEK 293T cells co‐transfected with Myc‐KIF2A, HA‐SMEK1, or scramble. E) CoIP analysis of HEK 293T cells co‐transfected with Myc‐KI2A and Smek1△Ran‐binding domain (RanBD)‐Flag or Smek1△DUF625‐Flag or Smek1△Arm‐Flag or Smek1△C‐term‐Flag. F) Proximity ligation assay of Myc‐KIF2A and HA‐SMEK1 in HEK 293T cell line. Magnified images indicated nuclear/perinuclear signals and cytosol signals. Scale bar, 20 µm. G) Immunofluorescent staining of Kif2a and Smek1 in primary cultured neurons. Scale bar in white, 20 µm; scale bar in yellow, 10 µm. H) Immunofluorescent staining of Kif2a in 2‐month cortex. Scale bar, 20 µm. I) SH‐SY5Y transfected with shSMEK1 and control vectors were treated with colchicine. Nuclear and cytosol fraction were isolated for western blot. J) QPCR analysis of KIF2A in 2‐month‐old mice cortices. N = 4 per group. K) QPCR analysis of KIF2A in 2‐month‐old mice hippocampus. N = 4 per group. Data are mean ± SD and are analyzed by two‐sided unpaired t‐test.

To identify the potential role of Smek1, we carried out protein mass spectrometry in SH‐SY5Y cells overexpressing Smek1 and the control group (**Figure** **6**A,B). STRING analysis of differentially expressed genes predicted a potential interaction between Smek1 and Kif2a involving proteins including RAN, RANBP1, ARFGAP1, ARFGAP2, and RANGAP1 (Figure 6C). Interestingly, ARFGAP1 and ARFGAP2 protein levels were increased in SMEK1‐overexpressing cells, while RANGAP1 levels were decreased. A coimmunoprecipitation (co‐IP) assay revealed the interaction between Kif2a, RAN, PPP4C, and Smek1 (Figure 6D). To map the Smek1 domain(s) required for interaction, we generated Smek1 mutants and assessed their interaction with Kif2a. The Smek1 N‐terminal deletion mutant (ΔRanBD mutant) failed to interact with Kif2a (Figure 6E), suggesting that the RanBD domain of Smek1 mediates Smek1's interaction with Kif2a. In the Duolink in situ proximity ligation assay (PLA), we identified ligation fluorescence in the cytoplasm and nucleus (Figure 6F). We thus speculated that the interaction between Kif2a and Smek1 takes place in both the cytosol and nucleus. Next, we aimed to investigate the distribution of Kif2a in the absence of Smek1. Costaining of Smek1 and Kif2a in primary neurons also revealed increased Kif2a in the cytoplasm of Smek1^−/−^ neurons (Figure 6G). In the cortex, we observed the deposition of cytoplasmic Kif2a in homozygous mice compared to wild‐type mice (Figure 6H). It has been reported that in mitotic cells, Kif2a is located in both the cytosol and nucleus in interphase and gradually aggregates into spindle microtubules in the nucleus during prophase to telophase.^[^ ^16^ ^]^ When fixed to metaphase, Kif2a was enriched in the cytoplasm in shSMEK1 cells compared to the control group (Figure 6I). qPCR of hippocampal and frontal cortex tissue showed no differences in the transcriptional level of KIF2A between homozygous and wild‐type mice (Figure 6J,K). Overall, these results indicated that Smek1 RanBD is responsible for interactions with Kif2a and that Kif2a tends to be restrained in the cytoplasm in the context of Smek1 depletion.

### 2.7 Depletion of Smek1 Triggered Apoptosis Through Mitochondrial Impairment

**Figure 7:**
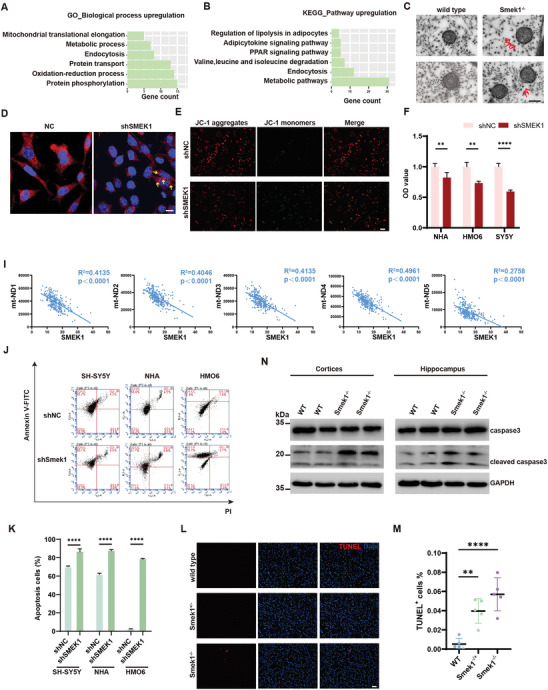
Smek1 deficiency caused abnormal mitochondrial transportation and function. A) Gene Ontology (GO) and B) KEGG analysis of upregulated proteins in SH‐SY5Y (SMEK1‐oe vs control group) proteomics. C) Electron microscope of sciatic nerve in 10‐month‐old mice. Red arrows pointed to microtubule disassociated to mitochondria. Scale bar, 0.2 µm. D) MitoTracker staining in SH‐SY5Y cells transfected with shSMEK1 and control vector. Yellow arrows pointed to aggregated perinuclear mitochondria. Scale bar, 20 µm. E) Transfected SH‐SY5Y cells were treated with lipopolysaccharide (LPS). Mitochondrial membrane potential was revealed by JC‐1 staining. Scale bar, 100 µm. F) Succinodehydrogenase activity was detected in transfected NHA, HMO6, and SH‐SY5Y cell lines. I) Correlation analysis of SMEK1 and mitochondria‐associated gene expressions in GTEx. J,K) Annexin V‐FITC and PI staining to assess apoptosis using flow cytometry in SH‐SY5Y, NHA, and HMO6 cells (transfected with shNC or shSMEK1) after LPS treatment. L,M) Cortical neuron apoptosis was detected by terminal‐deoxynucleoitidyl transferase mediated nick end labeling (TUNEL) in 12‐month‐old mice. Scale bar, 100 µm. N = 5 per group. (N) Western blot analysis of cortices and hippocampus in 12‐month‐old mice. Data are mean ± SD, and are analyzed by two‐sided unpaired t‐test; ^**^, *p* < 0.01; ^****^, *p* < 0.0001.

We aimed to investigate mitochondrial function, which largely relies on axonal transportation mediated by microtubule function. Analysis of the proteomic data showed that the upregulated proteins (SMEK1‐oe versus control group) were enriched in “oxidation‐reduction process”, “metabolic process”, “mitochondrial translational elongation” and “PPAR signaling pathway” (**Figure** **7**A,B). These results suggested that Smek1 might regulate mitochondrial function. To further investigate the effect of Smek1 on axonal transportation, we conducted sciatic nerve ligation. Sciatic nerve ligation analysis is a well‐established in vivo tool used to identify transported molecules in axons biochemically and immunohistochemically. Compared to the control group, Smek1 accumulated on the proximal side of the ligation site (Figure S2H, Supporting Information). To evaluate mitochondrial transportation, sciatic nerve was scanned by electron microscopy. As shown, mitochondria were disassociated from microtubules in Smek1^−/−^ mice, while wild‐type mitochondria were closely attached to the surrounding microtubules (Figure 7C). These results were further verified by MitoTracker staining. Compared to those in the control SH‐SY5Y cells, the mitochondria in shSMEK1 cells exhibited perinuclear aggregation (Figure 7D). JC‐1 staining showed lower mitochondrial membrane potential in shSMEK1 SH‐SY5Y cells (Figure 7E). The CCK8 assay in NHAs (astrocytes), HMO6 cells (microglia), and SH‐SY5Y cells showed lower cell viability in Smek1‐depleted cells (Figure 7F). Since mtDNA copies can partially reflect mitochondrial biogenesis triggered by injured mitochondria, we carried out transcriptome analysis based on the GTEx database. Interestingly, the results revealed consistent negative correlations between several mtDNA copies and SMEK1 expression levels in the frontal cortex (Figure 7I).

To assess whether mitochondrial impairment in Smek1 deficient cells could lead to neuronal loss, we next examined apoptosis levels. Smek1 knockdown caused significant apoptosis induced by LPS in SH‐SY5Y, NHA and HMO6 cells (Figure 7J,K). Terminal deoxynucleotidyl transferase (TdT) dUTP nick‐end labeling (TUNEL) staining of the PFC showed increased apoptosis in Smek1^−/+^ and Smek1^−/−^ mice (Figure 7L,M). Cleaved caspase3 was also increased in Smek1^−/−^ cortex and hippocampus tissue (Figure 7N). The above convergent findings indicated that abnormal mitochondrial transportation along microtubules in Smek1‐deficient mice resulted in mitochondrial impairment and caused cell death.

### 2.8 Tau Hyperphosphorylation and Mitochondrial Impairment could be Averted by Smek1 Overexpression and Kif2a Silencing

**Figure 8:**
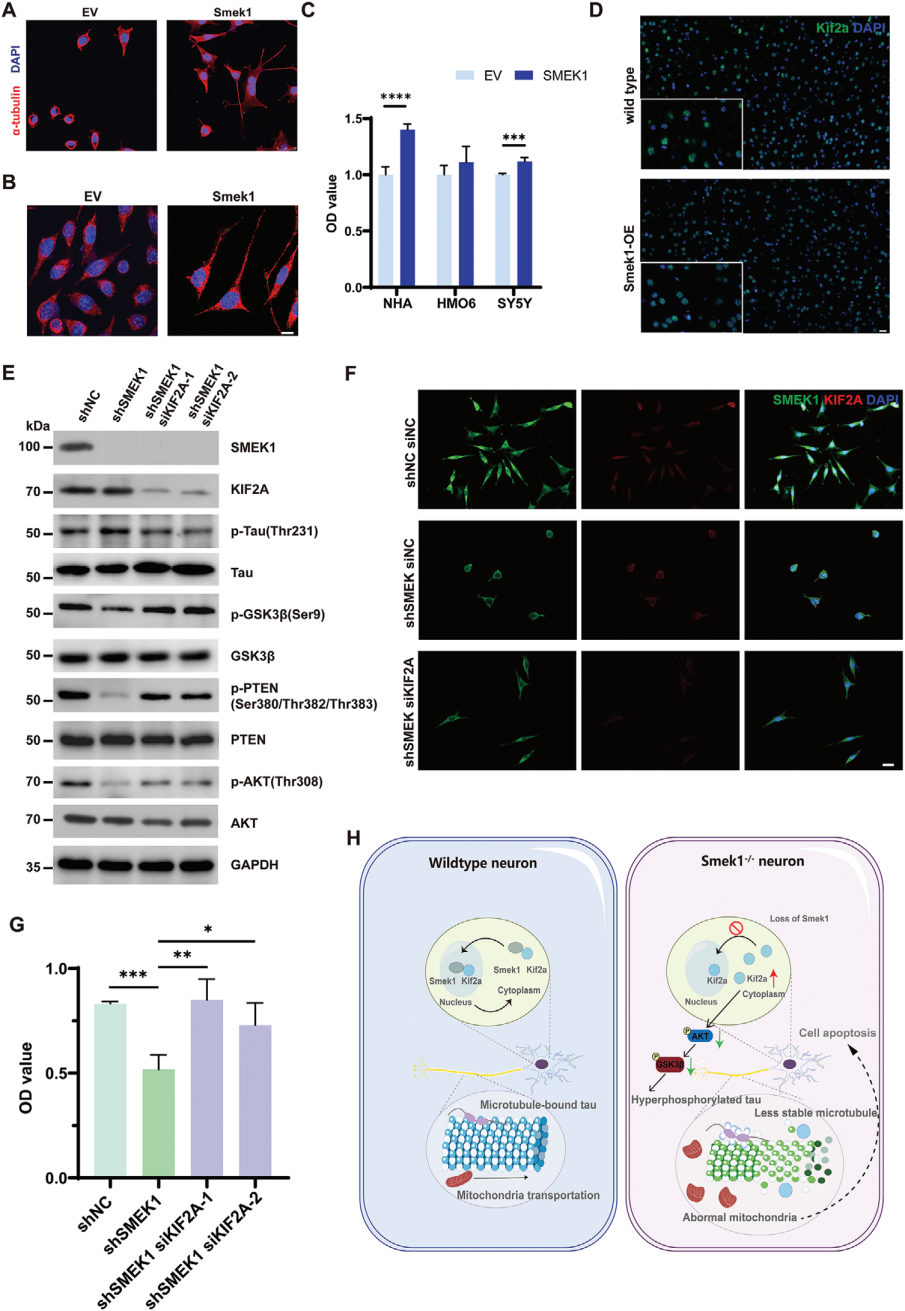
Tau hyperphosphorylation and mitochondria dysfunction could be averted by Smek1 overexpression and Kif2a silencing. A) Immunostaining of α‐tubulin in SMEK1 overexpression SH‐SY5Y cells. Scale bar, 25 µm. B) MitoTracker staining in SH‐SY5Y cells transfected with plvx‐SMEK1 and control vector. Scale bar, 20 µm. C) Succinodehydrogenase activity was detected in transfected NHA, HMO6, and SH‐SY5Y cell lines. D) Kif2a staining in 2‐month‐old cortex of Smek1 overexpressing mice and wild‐type. Scale bar, 20 µm. E) Protein levels of markers in AKT‐GSK3β pathways in SH‐SY5Y co‐transfected with shNC, shSMEK1, siNC and siKIF2A. F) Co‐staining of Smek1 and Kif2a in SH‐SY5Y cells co‐transfected with shNC, shSMEK1, siNC and siKIF2A. G) Succinodehydrogenase activity was detected in SH‐SY5Y cells transfected with shNC, shSMEK1, shSMEK1 with siNC and shSMEK1 with siKIF2A. H) Schematic view of Smek1 deficiency induces tau pathology via regulating Kif2a translocation. Data are mean ± SD and are analyzed by two‐sided unpaired t test; ^*^, *p* < 0.05; ^**^, *p* < 0.01; ^***^, *p* < 0.001; ^****^, *p* < 0.0001.

To illustrate the protective role of Smek1, we overexpressed Smek1 in SH‐SY5Y cells. In SMEK1‐overexpressing cells, we observed extended neurites (**Figure** **8**A), and mitochondria were evenly located in the proximal and distal cytoplasm (Figure 8B). The CCK8 assay in NHAs and SH‐SY5Y cells showed higher cell viability when Smek1 was overexpressed (Figure 8C). Kif2a was localized to the nucleus in Smek1‐overexpressing mice (Figure S3, Supporting Information), which was not different from that in wild‐type mice (Figure 8D). Interestingly, when Kif2a was silenced in shSMEK1 cells, western blotting revealed increased p‐GSK3β levels, which inactivated GSK3β and thus suppressed tau phosphorylation caused by Smek1 deficiency (Figure 8E). Immunostaining of tubulin revealed retained microtubules in siKIF2A shSMEK1 cells (Figure 8F). For mitochondrial function, the decreased cell viability in shSMEK1 could be rescued by knocking down Kif2a (Figure 8G). These results together confirmed that, through regulating the AKT‐GSK3β pathway, Kif2a is involved in neurodegeneration associated with the decreased expression of Smek1.

## 3 Discussion

In this study, we report that Smek1 was decreased in aged human brains. To analyze the functional significance of Smek1 deficiency in tau pathology progression, we generated Smek1 constitutive knockout mice. Loss of Smek1 resulted in substantial embryonic lethality as well as a shorter lifespan in mice. In middle‐aged Smek1^−/+^ and Smek1^−/−^ mice, we observed degenerative phenotypes characterized by decreased motor and cognitive abilities accompanied by gliosis and neuronal loss. Tau hyperphosphorylation was exacerbated, as detected by AT8, pThr231, and pSer396 immunostaining in the cortex, hippocampus, and striatum of Smek1^−/+^ and Smek1^−/−^ mice. These results indicated that Smek1 accelerated tau pathology progression even without a background of mutated tau.

Christopher et al. demonstrated a novel genome‐wide significant association between rs2273647‐T in *SMEK1* and cognitive decline in AD and mild cognitive impairment.^[^ ^6^ ^]^ They also conducted a follow‐up study and found a distinct variant in *SMEK1* associated with the risk of AD, which further confirmed the role of *SMEK1* in disease progression. Transcript expression analysis revealed that *SMEK1* is significantly lower in healthy controls than in AD patients in the temporal lobe but not in the dorsolateral prefrontal cortex. Moreover, healthy controls who carried rs2273647‐T had lower transcript expression in the temporal lobe, but the dorsolateral prefrontal cortex was exempted. Their findings suggested that increased *SMEK1* expression due to the rs2273647 genotype is protective in AD. In addition, we observed that Smek1 mRNA was significantly lower in healthy aged brains (prefrontal cortex and hippocampus) than in young brains, and that decreased expression of Smek1 in aged brains was associated with AD and the aging process. Single‐cell RNA sequencing confirmed that loss of Smek1 resulted in the generation of a novel neurodegenerative neuronal cluster in the murine brain. The reason for the contradictory conclusions between the previous study^[^ ^6^ ^]^ and our study is unclear; however, it is possible that region‐specific and disease‐specific effects caused varied results. Herein, we focused on the normal‐aged prefrontal cortex and hippocampus. The incidence of tauopathy is strongly related to age. AD primarily affects people older than 75,^[^ ^17^ ^]^ and primary age‐related tauopathy, in which tau deposition is directly correlated with cognitive deficits even in the absence of amyloid burden, is commonly observed in the brains of aged individuals.^[^ ^18^ ^]^ Given that tauopathy has been considered an essential hallmark of neurodegeneration and normal brain aging, such a reduction in Smek1 expression levels in aged brains may be the underlying mechanism of tauopathies that are due to age‐related effects.

Although *SMEK1* was initially reported as a significant single nucleotide polymorphism (SNP) that modifies cognitive decline in AD, we observed phenotypes other than dementia in a mouse model. Homozygotes displayed significant impairments in spatial memory, locomotor and sensorimotor deficits at 6 months, as demonstrated through rotarod, treadmill, T‐maze, and nest building tests. The deficits found in Smek1‐deficient mice are more likely to resemble symptoms in tauopathies other than AD, such as PSP and CBD. Hyperphosphorylation of tau and neurodegenerative pathologies in the cortex, hippocampus, and striatum are expected to contribute to the accelerated motor and cognitive phenotypes.

In the current study, we elucidated the underlying mechanism of microtubule abnormalities and mitochondrial transport in Smek1‐depleted neurons. In tauopathies, axonal loss may be ascribed to tau‐related or microtubule depolymerization protein‐mediated mechanisms.^[^ ^19^ ^]^ Kif2a, a member of the kinesin‐13 family, promotes microtubule disassembly, and hence, its hyperactivation could account for microtubule loss.^[^ ^20^ ^]^ In differentiated neurons, Kif2a mediates axonal degeneration by regulating microtubule disassembly and axonal breakdown during axonal pruning.^[^ ^21^ ^]^ In proliferating cells, Kif2a is distributed in the nucleus and cytoplasm during interphase, while aggregates of spindle microtubules and poles initiate prophase. At anaphase and telophase, Kif2a accumulates at the spindle midzone and midbody.^[^ ^16^ ^]^ Interestingly, the subcellular locations of Smek1 and Kif2a are remarkably similar. Smek1 is mostly located in the nucleus in interphase and prophase and moves to the cytosol and spindle in pro‐metaphase and anaphase.^[^ ^5d^ ^]^ Herein, we discovered that Smek1 may interact with Kif2a through its RanBD domain. The PLA assay displayed immunofluorescent signals in both the cytosol and nucleus, indicating that the interaction between Smek1 and Kif2a takes place in both structures. The binding of Kif2a and Smek1 may explain the common dynamic changes in these two proteins in mitotic cells. Moreover, an in vitro study revealed that silencing Kif2a reduced neural stem cell proliferation or self‐renewal but increased neuronal differentiation.^[^ ^22^ ^]^ Coincidentally, Smek1 promotes neuronal differentiation and suppresses the proliferative capacity of neural progenitor cells.^[^ ^5b,d^ ^]^ We believe that Smek1 and Kif2a are interdependent and intimately connected to one another in maintaining their biological functions.

Tau is a substrate of cyclin‐dependent kinase 5 (Cdk5) and Gsk3β. Sun et al. showed that Kif2a may regulate GSK3β activity via the β‐catenin‐AKT pathway.^[^ ^22^ ^]^ Here, we proved that Smek1 deficiency activated Gsk3β kinase through the AKT pathway and resulted in tau hyperphosphorylation. Knockdown of Kif2a in shSMEK1 cells reduced tau phosphorylation levels, indicating that increased cytosolic Kif2a in Smek1‐deficient cells may be responsible for tau hyperphosphorylation in an AKT‐Gsk3β‐dependent manner. On the other hand, axonal microtubule disassembly is largely dependent on the activity of Kif2a, the neuronal depolymerizing motor.^[^ ^20b^ ^]^ We speculate that Kif2a interferes with microtubule stability through 1) depolymerizing microtubule domains through energy‐dependent walking of the motor along the microtubule lattice and 2) regulating signaling pathways that lead to tau phosphorylation.

A previous study revealed prenatal death of Smek1 and Smek2 double knockout mice initiated at E14.5, while the number of Smek1 knockout mice throughout the embryonic period showed no abnormalities.^[^ ^5b^ ^]^ In contrast to previous observations, we herein report substantial prenatal death in Smek1^−/−^ embryos, which was manifested by deviation in Mendel's segregation rate. Previously reported Smek1/2 knockout mice were generated by gene trapping, and the knockout efficiencies of homozygous Smek1 were ≈45% at the mRNA level and 20% at the protein level.^[^ ^5b^ ^]^ In the current study, Smek1 was ablated to an efficiency of ≈20% at the mRNA level, and barely any protein remained in C57BL/6 mice. We speculate that the difference in lethality between Smek1 knockout mouse models is caused by differences in the expression of Smek1, since Smek1^−/+^ mice in this study which exhibited a comparable knockout efficiency to previous Smek1 homozygous mice, survived and lived for a normal lifetime. While Smek1^−/−^ mice are substantially embryonic lethal on the C57BL/6 background, the lethality of the Smek1^−/−^ mice was partially rescued in mice with a C57BL/6 and ICR mixed background, suggesting that the phenotypic consequence of Smek1 is greatly influenced by their genetic background. In C57BL/6 mice with conditional deactivation of the Smek1 gene in the nervous system, loss of Smek1 remains essentially remains lethal. We therefore speculate that the embryonic death of Smek1‐deficient mice is at least in part due to aberrant neurogenesis. Furthermore, we herein report that Smek1^−/−^ mice underwent unexpected death from 1.5 to 7 months of age. This result is in accordance with previous studies focusing on the function of SMK1/SMEK1 in *C. elegans* longevity.^[^ ^9^, ^10^ ^]^

There are several limitations in this study. First, complete loss of Smek1 caused substantial embryonic lethality and reduced body weight, which implies interference with murine development and growth. Analysis of human brain tissues showed a reduction in *SMEK1* expression in an age‐dependent manner. Thus, Smek1^−/−^ mice may not perfectly replicate the pathogenesis of tauopathies in aged humans. Nonetheless, partial loss of Smek1 in Smek1^−/+^ mice possibly recapitulated tau pathologies and the underlying mechanism. Second, while our study provided preliminary evidence that Smek1 deficiency may induce tau hyperphosphorylation and accelerate locomotor deficits in the nervous system, to date, no *SMEK1* variants or risk alleles have been identified in PSP, the tauopathy characterized as parkinsonism. A follow‐up analysis according to previous expression profiling of peripheral blood in dementia patients (GEO140830) revealed that *SMEK1* is also expressed at low levels in PSP patients (*p* = 0.0363). Although previous GWAS identified shared SNPs among various tauopathies and some variants participated in more than one cerebral pathological process,^[^ ^4^, ^23^ ^]^ further studies examining the biological function of *SMEK1* variants in tauopathy patients are warranted.

Taken together, these results demonstrated that Smek1 deficiency resulted in tau hyperphosphorylation and neurodegenerative phenotypes. Smek1 promotes Kif2a subcellular localization through protein binding, and Kif2a deposition in Smek1‐null cells causes microtubule disassembly by mediating the AKT/GSK3β/tau signaling pathway and further interferes with mitochondrial transportation along axons (Figure 8H). We conclude that Smek1 is essential for maintaining neuron viability; therefore, tauopathies may arise along with decreased Smek1 expression in the aged brain.

## 4 Experimental Section

### Microarray Dataset

Public human frontal cortical regions data from gene‐profiling dataset GSE53890 and human hippocampus data from gene‐profiling dataset GSE11882 were downloaded from Gene Expression Omnibus (GEO) using the Affymetrix Human Genome U133 Plus 2.0 Array platform.

### Animals

Smek1 knockout mice were generated as previously described.^[^ ^13^ ^]^ Smek1^flox/flox^ mice on a C57BL/6 background were generated with exon 2 flanked by loxP site.^[^ ^13^ ^]^ Concisely, heterozygous constitutive knockout mice (Smek1^−/+^) were generated by mating Smek1 floxed mice with Sox2‐Cre mice (Jackson Laboratories, stock No. 0 08454). Smek1^−/+^ mice were then self‐mated to generate Smek1^−/−^. Nervous system‐specific knockout mice were generated by mating Smek1 floxed mice with Nes‐Cre (Jackson laboratories, stock No. 0 03771) for several generations. Mice with C57BL/6‐ICR mixed background were generated by mating C57BL/6 constitutive knockout mice (Smek1^−/+^) with ICR wild‐type mice. Smek1^−/+^ mice with mixed background were then self‐mated. It should be noted that mice mentioned in this article referred to C57BL/6 strain unless otherwise stated. Genotyping was stated as previously described.^[^ ^13^ ^]^ All mice were bred and housed under specific pathogen‐free conditions. All experiments involving animals were approved by the Institutional Animal Care and Use Committee of Qilu Hospital of Shandong University (KYLL‐2021(KS)−870).

### Behavioral Assessment—Open Field Test

Male mice were used for open field test. In a dark and quiet room, mice were gently placed in a 40 × 50 × 50 cm methacrylate box and allowed to freely explore for 10 min and allowed. A video camera was attached above the arena and automatically recorded mice movements.

### Behavioral Assessment—Rotarod Test

Male mice at the age of 6‐month‐old and 10‐month‐old were used for the rotarod test. The Rotarod test was carried out in male mice. Animals were first submitted to training session (10 rpm for 600 s per day for 2 consecutive days). During the training session, animals should keep riding on the spinning rotarod for 600 s and were placed back to the rotating rod when fall off. After training session, animals were evaluated for six consecutive days, with the speed accelerating from 15 to 40 rpm for 900 s. Mice were placed on the spinning rotarod and the latency to fall off (duration time) was recorded. Animals staying more than 900 s were removed from the rotarod and their latency was recorded as 900 s. Mice were repositioned to the rotarod in the case of passive rotation. If this behavior was repeated, this was considered as the time to fall.

### Behavioral Assessment—Nesting behavior

Male mice at the age of 6‐month‐old were used for nesting behavior examination. Approximately 1 h before dark phase, mice were transferred to single cages containing 2.5 g pressed cotton material (50 cm × 60 cm). After 24 h, the presence and quality of nesting were rated ranging from 1 to 5 according to Deacon's criteria.^[^ ^15^ ^]^ 1 = nestlet not noticeably touched (more than 90% intact), 2 = nestlet partially torn (50–90% remaining intact), 3 = nestlet mostly shredded but often no identifiable nest site, 4 = an identifiable but flat nest, and 5 = a (near) perfect nest.

### Behavioral Assessment—Water T‐maze

Cognition ability of male mice at the age of 6‐month‐old were examined using water T‐maze. The T‐maze (length of stem 64 cm, length of arms 30 cm, width 12 cm, height of walls 16 cm) was made of clear plexiglass and filled with water (23 °C). A transparent platform (10 cm diameter) was placed in the target arm, 1 cm below the water level. The assay consists of an initial acquisition phase and reversal phase. During the acquisition phase, platform was placed on one side. Mice were placed in the starting area facing the back wall. Mice were given 30 s to complete each trial. If the platform was not found, mice were placed to the platform for 10 s. Mice were given 15 trials per day for 3 days. Correct choices were recorded for each mouse. On day 3, 20 min after the reinforcement, the platform was switched to the opposite arm, and mice were tested for the reversal phase. For each trial, in either initial acquisition phase and reversal phase, a correct choice was recorded if the mouse swam directly to the platform. If the mouse swam directly to the opposite arm, it was recorded as incorrect. If the mouse entered the target arm but swam back to the starting point before reaching the platform, it was recorded as “no choice”.

### Behavioral Assessment—Treadmill

Male mice at the age of 6‐month‐old were used for treadmill. Mice were trained at the speed of 9 m/min for 10 min on the first day and 11 m/min for 10 min (current value 1 mA) on the second day. During test phase, mice were placed in treadmill belt at the initial speed of 10 m/min (current value 1 mA) for 5 min. After 5 min, the speed was increased with 1 m/min acceleration. Mouse that was shocked for 6 consecutive seconds was regarded as “exhausted” and that time of exhaustion was then recorded. If the mouse were not exhausted for 1 h, the test was terminated and the exhaustion duration was recorded as 1 h.

### Primary Neuron Isolation and Culture

C57BL/6 mice cortices and hippocampus were dissected from E18.5 embryos, and the cells were mechanically dissociated by pipetting after carefully removing meninges and vessels. The samples were placed in precooled Hank's balanced salt solution (HBSS, Thermo Fisher) and were digested with 0.05% trypsin (Thermo Fisher). Cells were resuspended with DMEM/F12 containing 10% fetal bovine serum (Gibco FBS, Thermo Fisher) and growth medium (Neurobasal medium, Glutamax and B27 supplement, Thermo Fisher). Cells were centrifuged (80 g, 8 min) and plated onto poly‐D‐lysine (Sigma–Aldrich) at a density of 2 × 105 cells/cm^2^ at 37 °C with 5% CO2. For every three days, half amount medium was exchanged.

### Reverse Transcription and Real‐Time PCR

Total RNA extraction and real‐time PCR assays were performed as previously described.^[^ ^13^ ^]^ The expression of GAPDH was used as an internal control. The primers included the following:

**Table:** 

Name	Primers
SMEK1 (mus)	5′‐AACACTGCATACCAGAAACAACA‐3′ (Forward)
	5′‐CAGGTCCTGAGTGATGTCCA‐3′ (Reverse)
GAPDH (mus)	5′‐AGGTCGGTGTGAACGGATTTG‐3′ (Forward)
	5′‐TGTAGACCATGTAGTTGAGGTCA‐3′ (Reverse)
SMEK2 (mus)	5′‐ TATTGCGTAGTAACCGATTTCGC‐3′ (Forward)
	5′‐ GGTGTTATGACTGCCTTTCCTT‐3′ (Reverse)
KIF2A (mus)	5′‐ AATAGAGTGGTTGGTTCAGCAC‐3′ (Forward)
	5′‐ TGAAACGCTACCATTCTGTTGT‐3′ (Forward)
SMEK1 (homo)	5′‐TGAGAGCGACGGTTCTCTACT‐3′ (Forward)
	5′‐CACAATCAGAGTGTCCTGTTGTT‐3′ (Reverse)
GAPDH (homo)	5′‐CCAGGTGGTCTCCTCTGACTT‐3′ (Forward)
	5′‐ GTTGCTGTAGCCAAATTCGTTGT‐3′ (Reverse)

### Histology, Immunochemistry, and Quantification

For brain, spine, and sciatic nerve tissues, mice were anesthetized with 2% phenobarbital and perfused transcardially with sterile normal saline, followed by 4% paraformaldehyde (PFA). Dissected tissues were then fixed in 4% PFA. Fixed tissues were serially sectioned with 5 µm thick after paraffin‐embedding. After de‐wax and rehydrate paraffin sections, antigen retrieval was carried out using sodium citrate buffer, followed by washing in PBS. Following antibodies were used: SMEK1 (Sigma–Aldrich, HPA002568); GFAP (ServiceBio, GB11096); NEUN (ServiceBio, GB11138); IBA‐1 (ServiceBio, GB11105); MBP (Abcam, ab209328); p‐Tau (Thr231) (Abways, CY5625); p‐Tau (Ser396) (Abways, CY5657); AT8 (ServiceBio, GB113883); KIF2A (Sangon Biotech, D225969). The nuclei were stained with DAPI (Abcam, ab104139). Nissl's staining was performed with cresyl violet (Servicebio, G1036).

### FIJI

Image J software was used to analyze images For Smek1 and GFAP quantification, regions of interest (ROI's) of images were traced and transformed to RGB stack, images were thresholded to highlight positive staining, and the “Analyze Particle” function was used to obtain the percent area covered. For NeuN counting, regions of interest (ROI's) of images were traced and transformed to 8‐bit, images were thresholded to highlight positive staining, and the “Analyze Particle” function was used to obtain the count number. For Iba1 staining analysis, cells with increased Iba1 immunoreactivity together with an ameboid change in morphology were counted. Quantification was performed on five random fields per animal.

### Sciatic Nerve Ligation

Adult mice were anesthetized with 2% phenobarbital and the right sciatic nerve was ligated at the mid‐thigh level. Twenty‐four h after ligation, the mice were sacrificed, and 5 mm sections of the sciatic nerve proximal and distal to the ligation were isolated. As a control, the same length of the sciatic nerve at approximately the same location on the other side was also isolated.

### Electron Microscope

Mice were anesthetized with 2% phenobarbital and perfused transcardially with sterile normal saline, followed by fixation of 4% PFA/ 1% glutaraldehyde. Sciatic nerve and optic nerve were carefully isolated (length 1 cm), fixed in 4% PFA/2.5% glutaraldehyde. Samples were rinsed, 1% OsO_4_‐fixed, dehydrated and embedded in epoxy resin. Ultrathin (60–80 nm thickness) sections were stained with uranyl acetate‐lead citrate by standard procedures. Image J software was used to analyze microtubule counting. Regions of interest (ROI's) of images were traced and the number of microtubules was analyzed using “Cell Counter”. Quantification was performed on five random fields per animal in a blinded manner.

### Cell Culture, Lentivirus, and Cell Transfection

SH‐SY5Y (human neuroblastoma cell lines) were cultured in Dulbecco's modified Eagle medium (DMEM, Thermo Fisher) with 10% fetal bovine serum (AusGeneX, Australia), 1× GlutaMax (Thermo Fisher), penicillin and streptomycin (Thermo Fisher). NHA (human astrocytes) and HMO6 (human microglial cells) were cultured in DMEM with 10% fetal bovine, penicillin, and streptomycin. All cells were cultured in 5% CO_2_ at 37 °C.

Human full‐length SMEK1 plasmids were selected and inserted into the pLVX‐IRES‐Puro vector for stable overexpression. SMEK1 RNAi was designed to target the GATTTGTTTGCACAACTAA and ACTTGTATTGGAATTGTTA sequences in the GV248 vector. Lentivirus was packaged according to standard protocol: lentivirus plasmid of target gene or control plasmid was mixed with pPAX2 and pMD2.G in DMEM with lipo2000 (Invitrogen). The mixture was transfected into HEK293T cells and cultured in 5% CO2 at 37 °C. 3 h after transfection, culture medium was replaced by DMEM with 10% fetal bovine serum. After 48 h, draw the supernatant to a 15 mL centrifuge tube, filtered by 0.45 µm filter membrane, and stored in ‐80 °C refrigerator. Cells were transfected with short hairpin SMEK1 (shSMEK1) and pLVX‐SMEK1 RNA. CON077 and pVLX‐IRES‐Puro were transfected as negative controls. Cells were screened with puromycin at the appropriate concentration. The knockout efficiency was measured by qPCR (Figure S1F, Supporting Information) and shSMEK1‐2 was used for further studies. Overexpression of Smek1 in SH‐SY5Y was examined by western blotting (Figure S1G, Supporting Information).

To generate plasmids encoding Flag‐tagged Smek1 deletion mutants (ΔRanBD, ΔDUF625, ΔArm, or ΔC‐term), pCDNA3.1(+)‐H_PPP4R3A(p.1‐100del)−3×Flag‐C, pCDNA3.1(+)‐H_PPP4R3A(p.162‐355del)−3×Flag‐C, pCDNA3.1(+)‐H_PPP4R3A(p.356‐637del)−3×Flag‐C and pCDNA3.1(+)‐H_PPP4R3A(p.638‐820del)−3×Flag‐C was used.

pCMV3‐Myc‐KIF2A plasmid was purchased from Sino Biological Inc. pCMV3‐HA‐SMEK1 plasmid was constructed through PCR. For plasmid transfection, cells were seeded 24 h before transfection. Cells were then transfected by serum‐free medium with Lipofectamine 2000 (Invitrogen) according to the manufacturer's protocol and were replaced by fresh medium after 6 h.

SiKIF2A and negative controls were purchased from Genepharma (Shanghai, China). Sequences targeting KIF2A are listed below.

**Table:** 

Name	Sequence (5′‐3′)
si‐Nc	UUCUCCGAACGUGUCACGUTT
si‐KIF2A‐1	GGAUUUACGUGGAGAUCAATT
si‐KIF2A‐2	GGUGAUGUUCGUCCAAUAATT

### Western Blot Analysis

Tissues homogenate and cells were lysed by radioimmunoprecipitation assay lysis buffer (Bioteke) containing 1% protease inhibitor and 1% phosphatase inhibitors. Protein concentrations were determined by bicinchoninic acid method (Thermo Fisher). Samples were run in 10% Tris‐glycine gels and transferred onto PVDF membranes (Immobilon). The membranes were blocked in 10% semi‐fat dry milk in TBS (Tris‐buffered saline, 20 mm Tris pH 7.6, 150 mm NaCl) for 1 h at room temperature and incubated with primary antibodies overnight at 4 °C. After three rinses with TBS‐T (TBS supplemented with 0.05% Tween‐20) for 5 min each, the membranes were incubated with anti‐rabbit (Jackson ImmunoResearch, AB_2 338 504) or anti‐mouse (Jackson ImmunoResearch, AB_10 015 289) immunoglobulin conjugated with horseradish peroxidase. The membranes were rinsed with TBS‐T for three times, and were incubated with SuperSignal West Pico PLUS Substrate (Thermo Fisher). Primary antibodies included anti‐PI3K p85α (Affinity AF6241), anti‐p‐PTEN (Affinity, AF4450), anti‐PTEN (Proteintech, 60300‐1‐Ig), anti‐p‐AKT(T308) (Cell Signaling Technology, 13 038), anti‐p‐AKT(S473) (Cell Signaling Technology, 4060), anti‐AKT (Cell Signaling Technology, 4691), anti‐p‐GSK3β (Cell Signaling Technology, 5558), anti‐GSK3β (Cell Signaling Technology, 12 456), anti‐p‐Tau (Thr231) (Abways, CY5625), anti‐p‐Tau (Ser396) (Abways, CY5657), anti‐Tau (Abways, CY5022), anti‐KIF2A (Sangon, D225969), anti‐PP4C (Affinity, DF6833), anti‐Myc (Proteintech, 16286‐1‐AP), anti‐HA (Proteintech, 51064‐2‐AP), anti‐Flag (Sigma, F1804), anti‐Caspase 3 (Affinity, AF6311), anti‐cleaved Caspase 3 (Affinity, AF7022), anti‐Smek1 (for SH‐SY5Y, Abcam, ab70635), anti‐Smek1 (for tissue, Sigma–Aldrich, HPA002568). Antibody against GAPDH and β‐actin were from Abcam (Cambridge, UK).

### Immunoprecipitation Assays

HEK 293T cells were used for immunoprecipitation assays. Cells were washed with cold PBS and lysed by using cold IP lysis buffer (Thermo Fisher) at 4 °C by ultrasonography. Cellular extracts were incubated with appropriate primary antibodies with Dynabeads Protein G (10004D Invitrogen) on a rotator at 4 °C for 3 h. Beads were then washed three times with IP lysis containing cocktail, and the immune complexes underwent SDS/PAGE and then were immunoblotted with secondary antibodies.

### Immunocytochemistry

Adherent cells were cultured on cover slips and washed with 1 × PBS. Cells were then fixed and permeabilized. Nonspecific staining was blocked with 3% BSA for 1 h. Cells were incubated with primary antibody overnight at 4 °C. Primary antibody included anti‐Smek1 (Abcam, ab70635), anti‐Kif2a (Sangon Biotech, D225969), anti‐acetyl‐alpha tubulin (Affinity, AF4351), anti‐alpha‐tubulin (Affinity, T0033). After incubation with primary antibodies, cover slips were washed with 1 × PBS (3 × 5 min) and then incubated with appropriate secondary antibodies at room temperature for 1 h. For DUOLINK assay, cells were processed according to manufacturer's guidelines (Sigma–Aldrich). The nuclei were stained with DAPI (Abcam, ab104139).

### Bielschowsky Staining

Brain slices were incubated into 10% AgNO3 solution at 37 °C in dark for 30 min. The slices were washed with H_2_O followed by ammoniacal silver solution for 15 min. Developer solution (50% nitric acid + 0.8% formaldehyde + 0.2% citric acid in depH_2_O) was added and incubated for 10 min.

### Proteomics and Bioinformatics

We performed tandem mass tag quantitative proteomics in SH‐SY5Y cells (Smek1‐overexpressing vs control) and bioinformatics analysis to elucidate the underlying biochemical processes. Cells were subjected to appropriate sample preparation methods for MS‐based proteomics, including protein digestion, peptide labeling, fractionation, and MS analysis, and the raw data files obtained were processed with the MaxQuant search engine (v.1.5.2.8). DAVID Bioinformatics (version 6.8) was used to classify the differentially expressed proteins via Gene Ontology (GO) analysis, especially in the biological process category, and via Kyoto Encyclopedia of Genes and Genomes (KEGG) analysis. Protein interaction networks were generated using the STRING tool (https://cn.string‐db.org/).

### Cell Apoptosis and Flow Cytometry

For detection of apoptosis, a total of 1 × 10^5^ cells in each group were collected and suspended in 1× binding buffer and stained with Annexin V and PI (BD Biosciences). Fluorescence was detected by flow cytometry (BD Accuri C6 flow cytometer, BD Biosciences).

### Terminal Deoxynucleotidyl Transferase‐Mediated Nick End Labeling (TUNEL) Assay

For TUNEL analysis, a One Step TUNEL Apoptosis Assay Kit (C1090, Beyotime Biotechnology) was used and visualized. The nuclei were stained with DAPI (ab104139, Abcam), and apoptotic cells were labeled with cyanine 3 (Cy3).

### Cell Counting kit‐8 (CCK8) Assay

Cells (SH‐SY5Y, NHA, and HMO6) were planted in 96‐well plate with a density of 2 × 10^5^ cells per well. Each group as well as growth medium were planted in 6 wells for replications. After 6 h, each well was added with 10 µl CCK solution, incubated in 5% CO_2_ at 37 °C for 4 h and detected at absorbance of 450 nm.

### MitoTracker Staining

For staining cells that were to be fixed and permeabilized, 1 mm MitoTracker stock solution (Thermo Fisher) was diluted to 500 nm in growth medium. Cells were incubated with prewarmed staining solution in 5% CO_2_ at 37 °C for 30 min. After staining was complete, cells were washed with 1× PBS, stained with DAPI, and observed with Andor Dragonfly High‐Speed Confocal Microscope System. Data were analyzed with Image J FIJI.

### JC‐1 staining

Cells were cultured in 6‐well plate and incubated with 1 ml JC‐1 staining solution (Beyotime) and 1 mL growth medium in 5% CO_2_ at 37 °C for 30 min. After incubation, supernatant was removed. Cells were washed with 1×JC‐1 buffer twice and added with growth medium to maintain activity.

### Single‐Cell RNA Sequencing

Sample processing and sequencing quality control were previously described.^[^ ^13^ ^]^ In brief, Neocortex from both hemispheres and hippocampi from 2‐ month‐old mice (N = 2 for each group) were dissected and rinsed in DPBS. Tissues were then processed by using the Adult Brain Dissociation Kit (Miltenyi Biotec). Quality control and data processing were carried out by LC‐Bio Technology (Hangzhou, China). RNA‐seq datasets can be accessed on GEO: GSE171986.

### Statistical analysis

Data from mice and cell lines were presented as mean ± SD. Normality of data distribution was tested by using a Shapiro‐Wilks test. Student's t test was used to compare continuous data between groups (^*^p < 0.05, ^**^p < 0.01, ^***^p < 0.001, ^****^p < 0.0001). Two‐tailed χ^2^ test was performed on the number of mice genotypes. Kaplan‐Meier survival curves were generated and compared using the log‐rank test. Correlation analysis involved the use of linear regression and Pearson correlation coefficients. Statistical significance was considered at *p* < 0.05. The above analyses were carried out with GraphPad Prism 9. The detailed statistical analysis applied to each experiment is presented in the corresponding figure legends. The “N” of experiments in the figure legends represents biological replicates.

### Ethics Approval and Consent to Participate

All animal protocols were approved by the Institutional Animal Care and Use Committee at Shandong University School of Medicine.

## Conflict of Interest

The authors declare no conflict of interest.

## Supporting information

Supporting Information

## Data Availability

The data that support the findings of this study are available from the corresponding author upon reasonable request.
