# Supplementary material for: Loss of Smek1 Induces Tauopathy and Triggers Neurodegeneration by Regulating Microtubule Stability
Source: Adv Sci (Weinh). 2024 Aug 29;11(40):2400584. doi: 10.1002/advs.202400584 (PMC11516166; doi:10.1002/advs.202400584)
Supplement: Supplementary file 1 — Supporting Information [file ADVS-11-2400584-s001.docx]

Supplementary materials


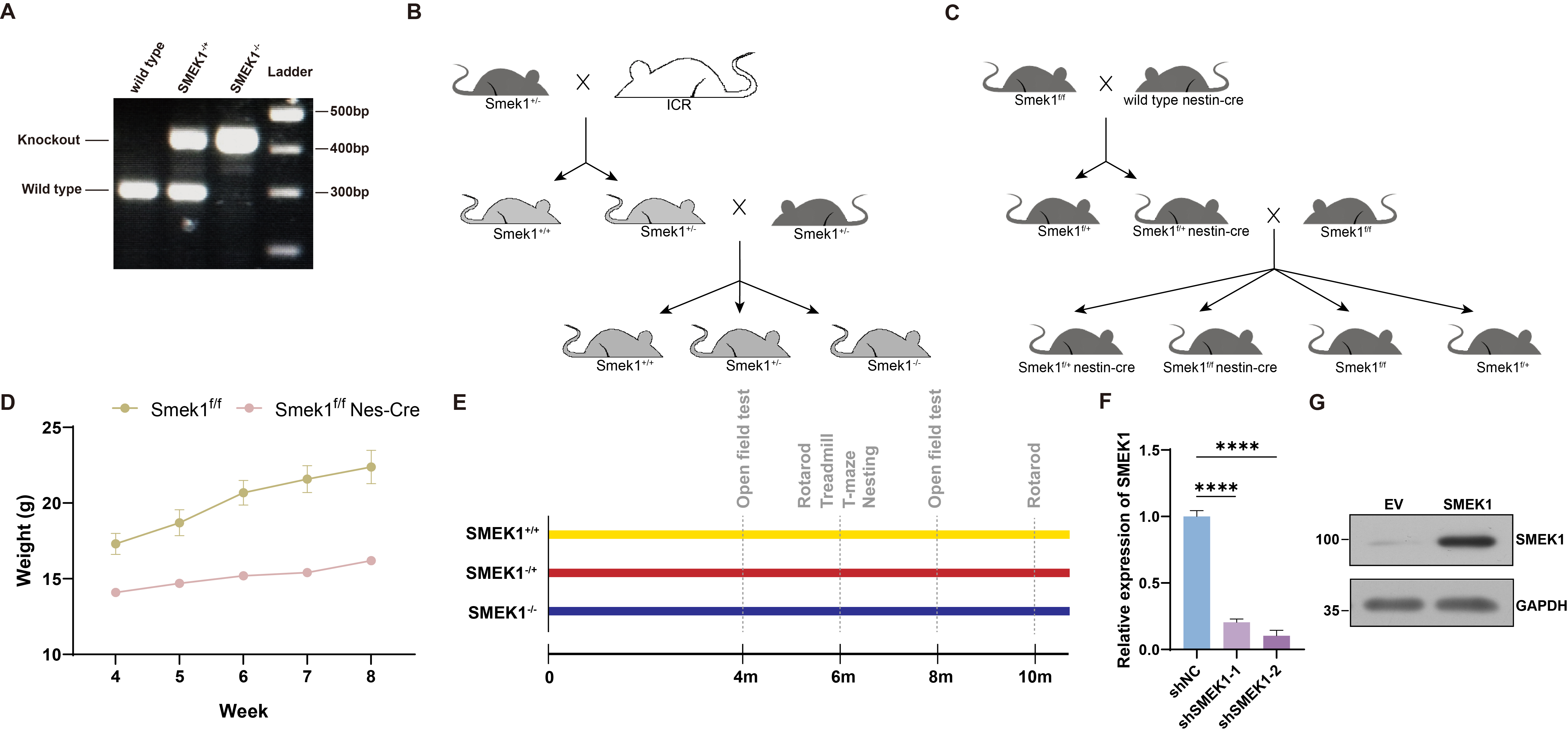


**Supplementary Figure 1**

(A) Mating strategy of C57BL/6-ICR mixed background Smek1 constitutive knockout (Smek1^f/f^ Sox2-Cre) mice. (B) Genotype of ICR-C57BL/6 Sox2-Cre knockout mice. PCR products were amplified with F1, R1 and R3 simultaneously. (C) Mating strategy of Smek1 nervous system conditional knockout (Smek1^f/f^ Nes-Cre) C57BL/6 mice. (D) Body weight of Smek1^f/f^ Nes-Cre between 4-week-old to 8-week-old (N=5 for Smek1^f/f^ mice and N=1 for Smek1^f/f^ Nes-Cre mice). (E) Experimental schematic of the behavior tests. (F) Knockout efficiency of shSMEK1 in SH-SY5Y cells. (G) Western blot of SH-SY5Y cells transfected with pLVX-IRES-Puro empty vector and pLVX-SMEK1. Data are presented as mean ± SD; ****, P < 0.0001.


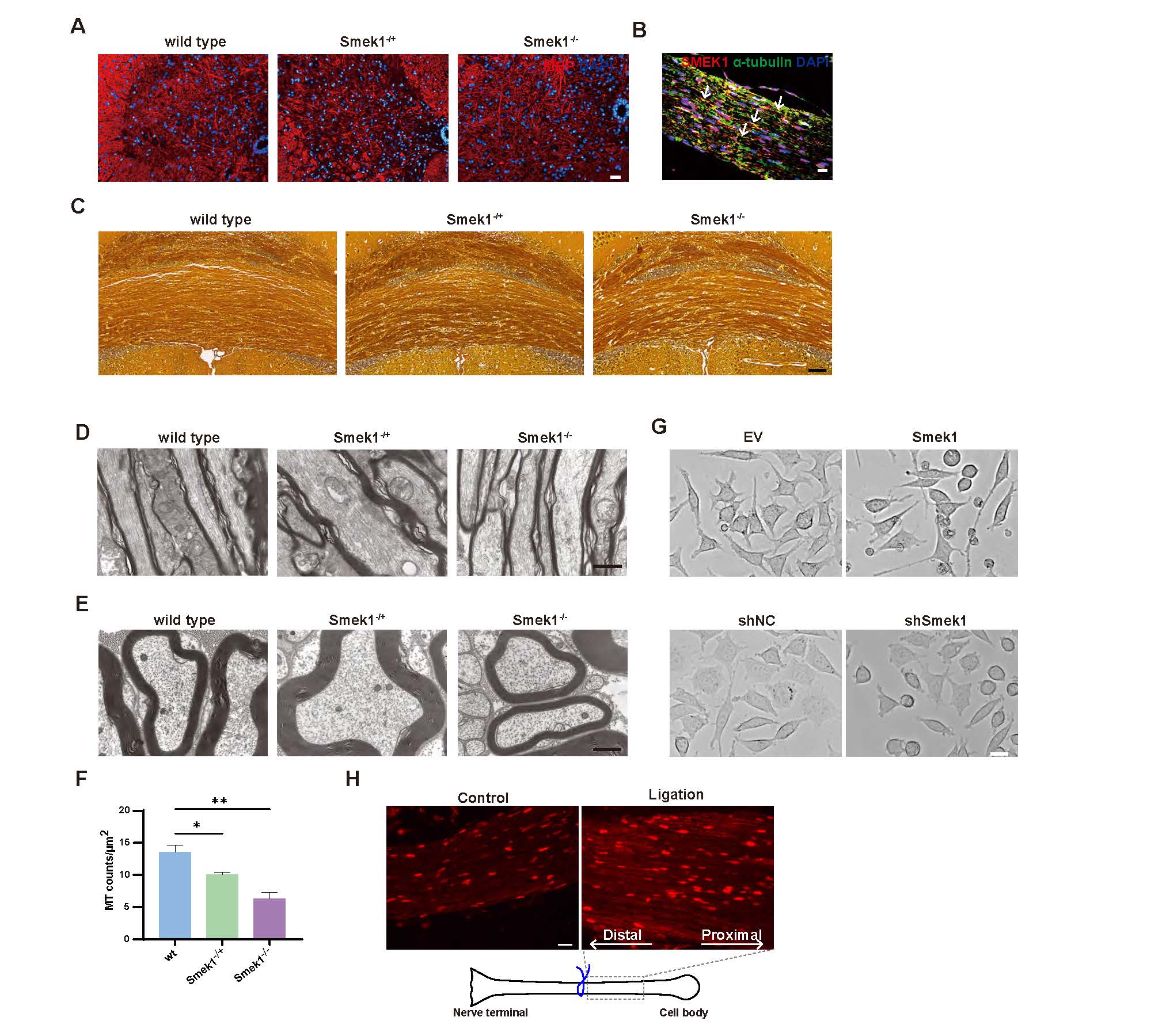


**Supplementary Figure 2**

(A) Immunofluorescent staining of MBP in spinal cord. Scale bar, 20 μm. (B) Co-staining of SMEK1 with microtubule marker α-tubulin in sciatic nerve. Scale bar, 20 μm. (C) Bielschowsky silver staining of corpus callosum in 12-month-old brain. Scale bar= 50 μm. (D) Electron microscopic image of vertical section of optic nerve in aged mice. Scale bar, 1 μm. (E) Cross section of sciatic nerve under electron microscope. Scale bar, 1 μm. (F) Microtubule density counted in sciatic nerve cross sections (n=4 in each group). (G) Live cell imaging of SH-SY5Y cells transfected with SMEK1 vector, shSMEK1 and corresponding empty vector. Scale bar, 20 μm. (H) Immunofluorescent staining of Smek1 in control and ligated sciatic nerve. Scale bar, 50 μm. Data are mean ± SEM and are analyzed by two-sided unpaired t test; *, P < 0.05; **, P < 0.01.


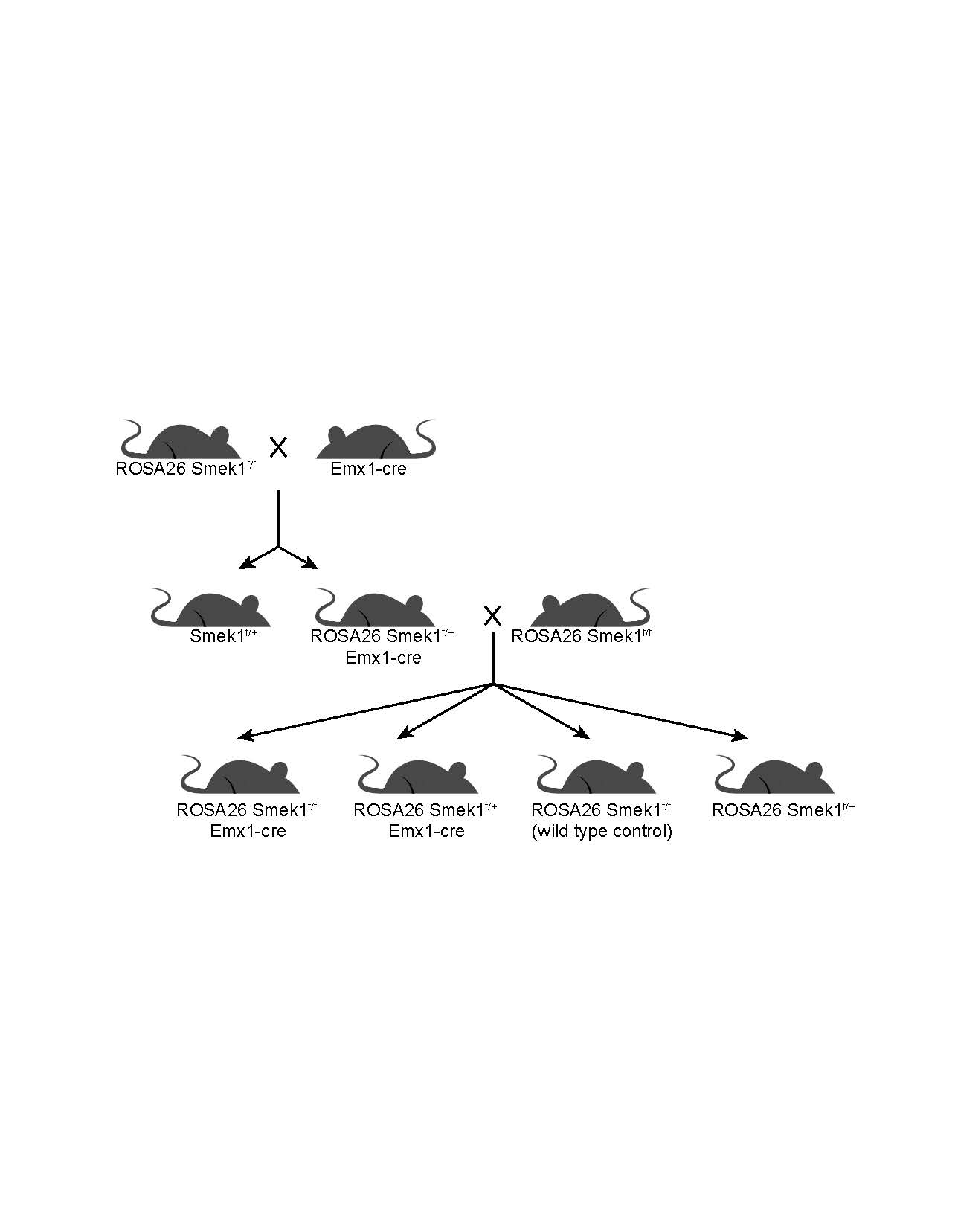


**Supplementary Figure 3** Mating strategy of Smek1 conditional overexpressing mice (ROSA26-Smek1^f/f^ Emx1-cre)
